# The Impact of Water Table Drawdown and Drying on Subterranean Aquatic Fauna in In-Vitro Experiments

**DOI:** 10.1371/journal.pone.0078502

**Published:** 2013-11-20

**Authors:** Christine Stumpp, Grant C. Hose

**Affiliations:** 1 Institute of Groundwater Ecology, Helmholtz Zentrum München, Neuherberg, Germany; 2 Department of Biological Sciences, Macquarie University, Sydney, New South Wales, Australia; The Ohio State University, United States of America

## Abstract

The abstraction of groundwater is a global phenomenon that directly threatens groundwater ecosystems. Despite the global significance of this issue, the impact of groundwater abstraction and the lowering of groundwater tables on biota is poorly known. The aim of this study is to determine the impacts of groundwater drawdown in unconfined aquifers on the distribution of fauna close to the water table, and the tolerance of groundwater fauna to sediment drying once water levels have declined. A series of column experiments were conducted to investigate the depth distribution of different stygofauna (Syncarida and Copepoda) under saturated conditions and after fast and slow water table declines. Further, the survival of stygofauna under conditions of reduced sediment water content was tested. The distribution and response of stygofauna to water drawdown was taxon specific, but with the common response of some fauna being stranded by water level decline. So too, the survival of stygofauna under different levels of sediment saturation was variable. Syncarida were better able to tolerate drying conditions than the Copepoda, but mortality of all groups increased with decreasing sediment water content. The results of this work provide new understanding of the response of fauna to water table drawdown. Such improved understanding is necessary for sustainable use of groundwater, and allows for targeted strategies to better manage groundwater abstraction and maintain groundwater biodiversity.

## Introduction

Groundwater fauna (stygofauna) are generally the highest organisms of groundwater ecosystems. They are important contributors to the health of the ecosystem by maintaining water quality and water flow in aquifers, and processing organic matter [1], [2]. To date, most research on stygofauna has focused on the diversity and phylogeography of the fauna (e.g., [3]) with relatively little focus on ecology [4]. In particular, little is known about the functional role of fauna in the aquifer, how the fauna is influenced by changes in the hydrology of groundwater systems or how well they can cope with environmental changes. In this study, we examine the behaviour of stygofauna in the vicinity of the water table, how they respond to declining water tables and if they can potentially survive in the unsaturated zone after water tables have declined.

Groundwater ecosystems may be vulnerable to environmental change and disturbance due to the relatively stable physicochemical conditions in the aquifers to which fauna are adapted [5]. It is known that microorganisms are strongly adapted to such stable conditions and any environmental change might lead to changes in community structure [6]. Stygofauna may be similarly sensitive [7].

Within groundwater, there is often a natural gradient of decreasing oxygen and nutrients with depth below the water table [8]. Consequently, stygofauna are often most abundant and diverse at shallow depths, closer to the groundwater table, and richness and abundance generally decrease with distance below the water table [9], [10]. Fluctuations in the groundwater table, particularly declining water tables, may therefore, greatly influence stygofauna assemblages.

Global demand for water is placing increasing pressure on aquifers as a reliable source of potable water [11]. The impact of water abstraction and declining water tables is particularly pronounced in shallow aquifers, which also tend to support the most diverse and abundant fauna assemblages [9], [10]. Overexploitation (e.g. for drinking water, irrigation or mining purposes) is considered to severely impact groundwater ecosystems (e.g., [12]), and changes in rainfall and recharge associated with climate change also result in declining water tables, albeit over longer time scales.

The understanding of ecosystem functioning in the vicinity of the water table is crucial as this highly reactive and complex zone may also function as an important buffer and filter. It likely plays a crucial role in dynamic natural attenuation processes [13]–[16], which requires both, a deep understanding of the hydrogeological environment as well as its biological nature. At boundaries - like the hyporheic zone - it is known that ecotones have developed regulating the transfer of nutrient and matter fluxes across compartments [6], [17], [18]. Similarly, the zone of water table fluctuations (i.e. the boundary between unsaturated and saturated zone) may result in an ecotone with different communities and diversity compared to the constantly saturated and constantly unsaturated zones. It is expected that biota can cope for a certain time with water stress (e.g., [19]) and are adapted to dynamic, altering nutrient and water content conditions. However, any water table variations, which are more pronounced than under normal fluctuating conditions, are suggested to be stressors not only on terrestrial ecosystems but also on the groundwater ecosystem itself [20]. It is suggested that permanent changes in the depth of water levels have even more drastic impacts on ecosystem functioning. Fauna, particularly those restricted to live in larger pores, will be affected immediately by water table decline as the larger pores drain first while water may remain in smaller pores for longer. Nevertheless, the mobility of fauna will be impaired by draining and disconnecting pore regions from the main flow paths.

The decline of the groundwater table due to reduced groundwater renewal or abstraction generally reduces the volume of accessible habitats or disconnects groundwater from surface waters [21]. Rapid declines in groundwater may lead to stranding of organisms in layers above the water table [19]. Still, it remains unknown whether organisms can generally cope with the decline, survive in zones with decreased water contents and/or follow the declining water table depending on abstraction rates and volumes. Current understanding of the impacts of pumping and groundwater abstraction on biodiversity is limited [22], largely speculative and guided by a small number of correlative studies [23], [24]. From irrigation water withdrawal in rivers, impacts on invertebrates were indirectly identified through changes in the physicochemical environment as well as directly through reduced flow which was intensified by the magnitude and duration of withdrawal [25]. Recognising the need for ongoing abstraction worldwide, protection of biodiversity in groundwater ecosystems may be achieved by considered management of how and when groundwater is abstracted.

The objective of this study was to identify the high-resolution spatial distribution of groundwater fauna below the water table and how the fauna cope with different rates of water table declines. Further, the survival of organisms under various saturation conditions was investigated. Column experiments were performed to investigate depth distribution of different stygofauna under saturated conditions and after two different rates of water table declines. The survival rate of the organisms was tested in desiccation experiments. The results of this work will close research gaps of understanding implications of hydrological changes to the groundwater ecosystem. This improved understanding is mandatory for a sustainable use of groundwater and allows for targeted adaptation strategies such that the future abstraction and management of groundwater can be operated to mitigate impacts on groundwater biodiversity.

## Materials and Methods

### Sampling sites

Stygofauna and groundwater were collected from two different aquifers. Copepoda (Cyclopoida: Cyclopidae) and Syncarida (Parabathynellidae) were collected from a porous, alluvial aquifer associated with the Macquarie River at Wellington, NSW, Australia (32°34′S, 148°59′E). Samples were collected from a shallow bore (WRS05) at the University of NSW hydrological research station. The bore was 23 m deep, with a screened section between 15–18 m below ground surface. Groundwater levels closely follow water levels in the adjacent river. During base flow conditions water level is approximately 12 m below ground surface but may rise up to 3 m below ground surface during flood events (Peter Graham, UNSW, pers. comm., unpub. data). The bore intersected the shallow, unconfined, quarternary alluvial aquifer, and at this location, the aquifer has considerable hydrological exchange with the adjacent river. The alluvium and units within it vary in size from silts and clays to gravel and cobble units. The area has a diverse stygofauna, including copepods, syncarids [26] and ostracods [27].

Copepoda (Cyclopoida: Cyclopidae) were also collected from an upland swamp in the Budderoo National Park, NSW, Australia (34°37′S, 150°40′E). This site is located approximately 600 m above sea level and about 30 km south west of Wollongong in the headwaters of the Kangaroo River. The sediment of the swamp comprises basal layers of coarse sands, overlain by several layers of organic accumulation up to 3.3 m in thickness. The water table varies between ground surface and about 0.3 m depth below ground surface in the central swamp area. Here, the fauna was collected with bailers from fully screened piezometers up to 2 m in depth. The fauna of the swamp is dominated by copepods but includes syncarids, amphipods and ostracods [28]. Fauna were collected from Budderoo National Park under NSW NPWS collection permit No SL100800.

The fauna are all undescribed species. In the absence of taxonomic keys for most stygofauna, the mitochondrial Cytochrome Oxidase 1 gene was used to identify the taxa. Methods for this analysis are described in detail in Asmyhr and Cooper [27]. Resulting Genbank accession numbers are KF361321 - KF361324. Both taxa collected from Wellington were considered stygobytic in that they lacked pigmentation and eye spots. The copepods from the swamp were considered stygophilic. Although lacking pigment, they did have an eye spot. Molecular analyses of multiple specimens of each taxon from each location indicated that they were each a single taxonomic unit ([26], Asmyhr & Hose unpub. data). Our use of taxa other than syncarids and copepods in experiments was limited by low abundances in collected samples.

### Column Experiments

Column experiments were conducted to investigate high resolution depth distributions of stygofauna and the impact of fast and slow water table drawdown on these depth distributions. Separate experiments were performed with fauna from different sites using groundwater from the collection sites. The acrylic glass columns were 160 mm tall and had a diameter of 50 mm. They were filled with commercially available glass beads (1.55–1.85 mm), giving a particle density δ_s_ of 2.5 kg/L. Uniform glass beads were chosen (i) to maintain reproducible conditions of the pore system between different column experiments which cannot be ensured using heterogeneous, natural sediments; (ii) to create a uniform pore size distribution throughout the entire length of the column; (iii) to restrict migration of organisms due to pore size changes alone; (iv) to avoid pore size exclusions; (v) to actually track bigger organisms visually; and (vi) to increase the recovery rate of organisms by avoiding any fine material (<size of organisms) which would result in difficulties counting the fauna under the microscope.

For the experiments, the columns were filled with oven-dried glass beads and groundwater in a stepwise manner. The columns were filled to half way (8 cm) before the test organisms were added. Test animals were placed into the middle of the column (8 cm). The columns were then carefully filled with glass beads and groundwater to the top (16 cm). Capillary rise was minimized during the filling procedure to ensure that the organisms remained in the middle of the column by minimizing excess water before adding additional glass beads.

Either 20 Copepoda or nine Syncarida were used in each column, and all treatments had three replicate columns. Animals were randomly allocated to treatments. All experiments were conducted in environmental cabinets under conditions approximating those in groundwater (T = 18°C, darkness). We conducted preliminary trials to compare the distributions of animals added to the top (after filling) and to the middle of the column. There was no obvious difference on fauna depth distribution when fauna were added to the top or middle of the columns.

**Figure 1:**
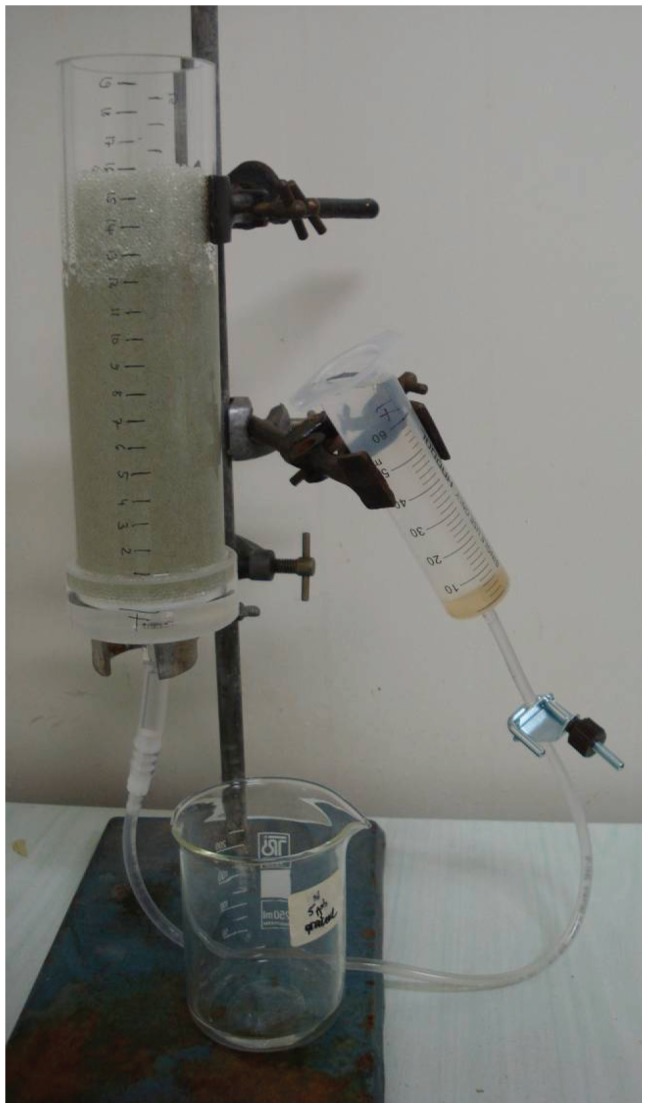
Experimental setup investigating the impact of drawdown on stygofauna. Column filled with glass beads and with water table lowered to 8; note the unsaturated zone in the top 4 cm and the apparently saturated capillary fringe from 8-4 cm; Photo C Stumpp.

After an acclimation period of 48 h, the stygofauna depth distributions were investigated under three different hydrological conditions. First, the columns remained saturated and water level was not changed. These columns served as a control. Second, the water table was rapidly drawn down to the middle of the column leading to relatively higher decline rates (2.6 m/d) during drainage which lasted 0.25 h. Third, the water table was slowly drawn down to the middle of the column in a step wise manner, leading to relatively slower decline rates (1 m/d) during drainage which lasted 2 h. For the two drawdown treatments, the water level inside the column was controlled by moving a connected water reservoir downwards (Fig. 1). Draining of organisms was restricted by using a porous plate (100–160 µm) and a fine filter (Whatman filter paper No 1) at the bottom of the column. After the drawdown, the water connection between the column and the reservoir was closed. Sediment was removed from the columns with a spoon in 1-cm steps and collected in separate beakers together with excess water which was removed with a pipette. Due to inaccessibility, the last two layers (0–2 cm) in the column were collected and analysed together. The sediment was sieved (1 mm) and flushed to separate the stygofauna from the glass beads. Subsequently, the fauna was counted under the microscope. According to the hydrological conditions, we compared three different depth groups, i.e. number of organisms below the water table (0–8 cm), in the capillary fringe (8–12 cm) and in the unsaturated zone (12–16 cm)

The mass of the glass beads (M) added to the column was recorded to determine the bulk density (δ_b_) and the porosity (n) considering the total volume of the column (V_tot_) and the particle density (δ_s_):
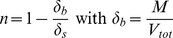
(1)The saturated hydraulic conductivity of the glass beads was determined in the packed columns with a constant head method (n = 18). The saturated hydraulic conductivity, and also the hydraulic gradient, governs the water flow rate of the declining water table.

Further, draining water from the drawdown experiments were weighed to determine the mass and volume of water loss (V_D_) and thus the mean water content (θ) above the water table (16-8 cm):
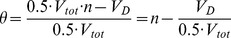
(2)


### Desiccation Experiments

Desiccation experiments were conducted to investigate the viability of fauna in sediments with different water contents. These experiments give information on the potential survival of organisms stranded in the unsaturated zone due to water table draw down. Petri dishes (H = 12 mm, ∅ = 50 mm) were filled with glass beads and groundwater yielding similar porosities as in the column experiments. Ten Copepoda or five Syncarida were added and acclimated to test conditions for 24 h. The dishes with fauna were left to evaporate at 18°C in a dark environmental cabinet until a specific saturation level was reached, as determined by the mass of water lost from the dish. Once the appropriate moisture content was reached, the dishes were covered, sealed with parafilm, and left for 48 h in the dark environmental cabinet at 18°C. Three replicate dishes were used for each saturation level. Animals were randomly allocated to treatments. The saturation levels used were 100 (total saturation), 66, 33, 16.5, 8.25, 4, 2, and 0% (no water remaining). At the end of the experiment the petri dishes were resaturated and the number of surviving animals was recorded. Death was recorded when the animal failed to respond within 15 s of gentle stimuli. This test for death was done twice, first at the time of sample processing, and again later after all dishes had been analysed. The saturation levels at which 10, 50 and 90% of the organisms were dead (Lethal Saturation Level 10 (LSL_10_), Lethal Saturation Level 50 (LSL_50_), and Lethal Saturation Level 90 (LSL_90_)) were estimated based on the mortality data.

### Statistical Analysis

The distribution of the animals within the columns was described using a cumulative distribution function. The proportions of animals at each depth were added cumulatively as depth decreased (i.e. starting at the bottom of the column, 0 cm). The cumulative distributions were described by fitting a three parameter, non-linear regression function, with a binomial error structure and a fixed upper limit of one using the DRC package [29] in R version 2.15.2 [30]. Several models were fitted to the data, with the most appropriate chosen as that which had the lowest Akaikes information criterion (AIC) value. The model parameters were estimated using maximum likelihood, with starter values determined by the programs self-starter function. The DRC package allows multiple curves to be fitted simultaneously and makes it possible to compare curves from independent tests using mixed-model non-linear regression analysis [31]. The method assumes that for any particular concentration response curve, the parameters are fixed but may vary from test to test due to some random effect. That is, the parameters for any test represent the ‘average’ parameters for that curve plus or minus a test-specific error. In this way, including both fixed and random effects in our non-linear regression model makes it possible to account for variation among treatments. Thus, the full non-linear regression model is one that includes random effects, i.e., individual parameters are determined for each individual curve.

Under a null hypothesis of no difference between two or more curves, the parameters of the curves can be considered to be drawn from the same population, i.e., the random effects that represent the inter-test variability should be negligible. Therefore, replacing the individual parameters with a common parameter (thereby removing the random effects) should not affect the residual deviance of the non-linear model. The test for reduction of a full model (with random effects) to a simpler model (without random effects) can be tested using a likelihood ratio test [31].

Further, we compared between treatments the proportions of fauna remaining in the top 4 cm of each column at the end of the experiments, which was the zone of unsaturated conditions above the capillary fringe. This analysis was done using a generalised linear model with a logit link and binomial error structure, using the glm function in R version 2.15.2 [30]. Assuming that animals in the columns move downward with the receding groundwater level and not get strained in the unsaturated parts, we expected that the proportion of fauna in the upper levels of the drawdown treatments would be less than that in the fully saturated columns (controls). Accordingly, we tested the specific hypothesis that the proportion of animals in the unsaturated treatments will be less than that in the control columns.

The mortality of animals in the desiccation experiments was analysed using non-linear regression, with a binomial error structure using the DRC package [29] in R version 2.15.2 [30]. The model choice was based on the AIC as described above. The lethal saturation level (LSL) at which 50% (LSL_50_), 90% (LSL_90_) and 10% (LSL_10_) of the population died were extrapolated from the fitted curve.

## Results

### Column setup

The porosity was determined in every column and was on average 0.37±0.01 m^3^/m^3^. The saturated hydraulic conductivity was tested in six columns three times each. The average saturated hydraulic conductivity (±SD) of the columns was 1.8 (±0.3)×10^−3^ m/s. The small standard deviations of both parameters indicate reproducible pore structures and hydraulic properties in all experiments. Thus, the hydrological conditions of all experiments are similar and the results are not affected by the experimental setup.

Drawdown of the water table by 8 cm resulted in a saturated zone in the bottom 8 cm of the column, a zone in which the capillary fringe caused saturation of the sediment for a height of 4 cm above the water table, and an unsaturated zone in the top 4 cm (see Fig. 1). After drawdown, the average water content above the water table was 0.24±0.02 m^3^/m^3^ in the top 8 cm and 0.12±0.05 m^3^/m^3^ in the top 4 cm considering that the capillary fringe is completely saturated. This equals a saturation level of 65% and 32%, respectively.

### Drawdown experiments

#### Syncarids

**Figure 2:**
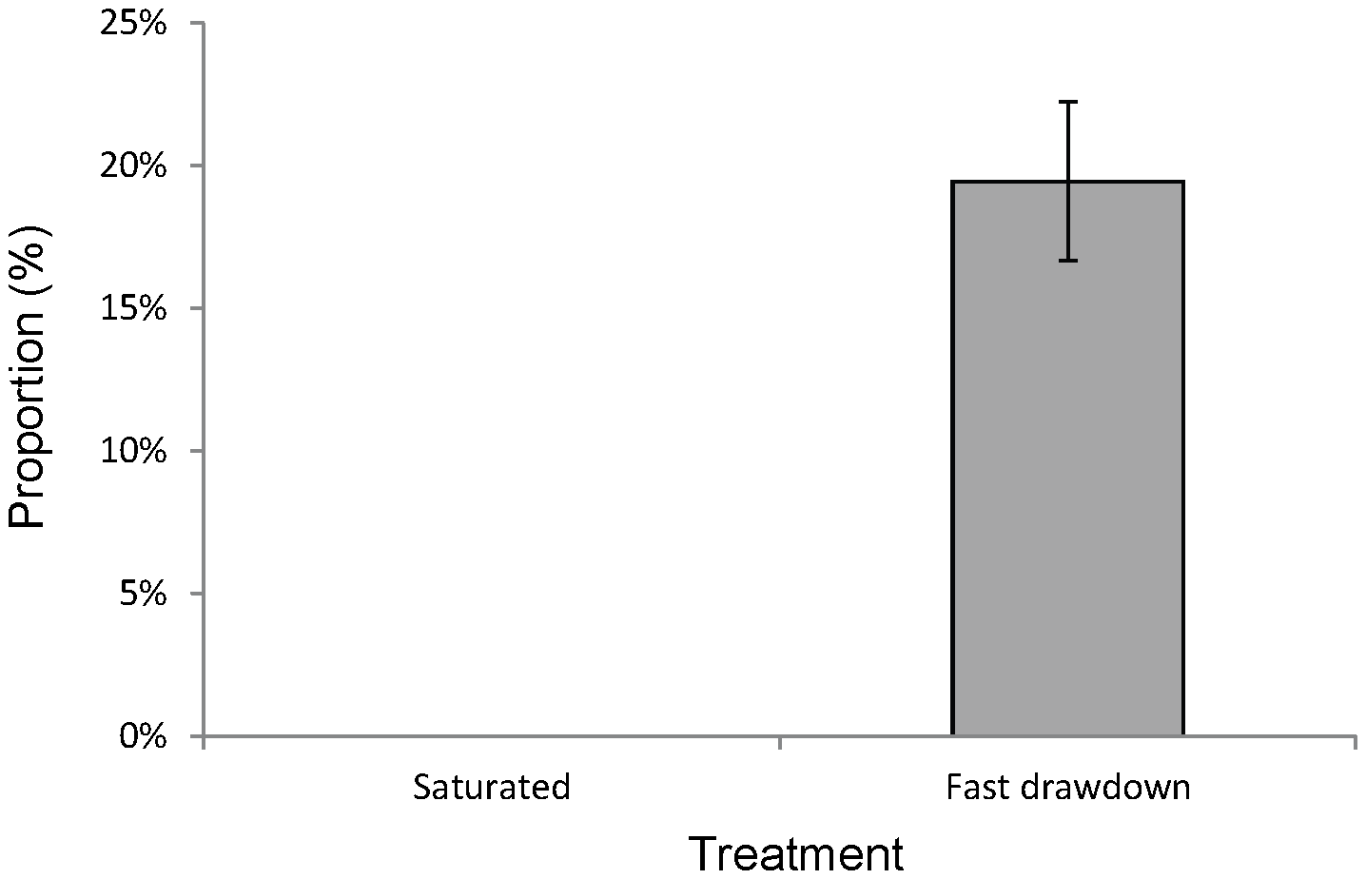
Mean (±95% CI) proportion of syncarids in the top four centimetres of the sediment. Comparison of results on mean proportion of syncarids in the saturated and fast-drawdown columns; in the latter the top four centimetres represents the unsaturated zone in the fast drawdown columns and shows the proportion of animals stranded above the water table as the water level is lowered.

All syncarids in the saturated treatments were collected from the bottom 2 cm of the column, suggesting a propensity for this taxon to burrow deeply. In the fast drawdown treatment, 75% of the syncarids were found below the water table (0–8 cm) and some in the capillary fringe (6%; 8–12 cm). However, 19% of the syncarids remained in the unsaturated part of the column (12–16 cm; Fig. 2).

There was a significant difference in the distribution of the syncarids among treatments, with a greater proportion of syncarids found at shallower depths (higher in the column, closer to the water table) in the drawdown columns than in the controls. Importantly, however, the proportion of syncarids in the unsaturated zone of the drawdown treatment (12–16 cm) was significantly greater (p = 0.004) than (and specifically, not less than) that in the saturated treatment.

#### Wellington Copepods

**Figure 3:**
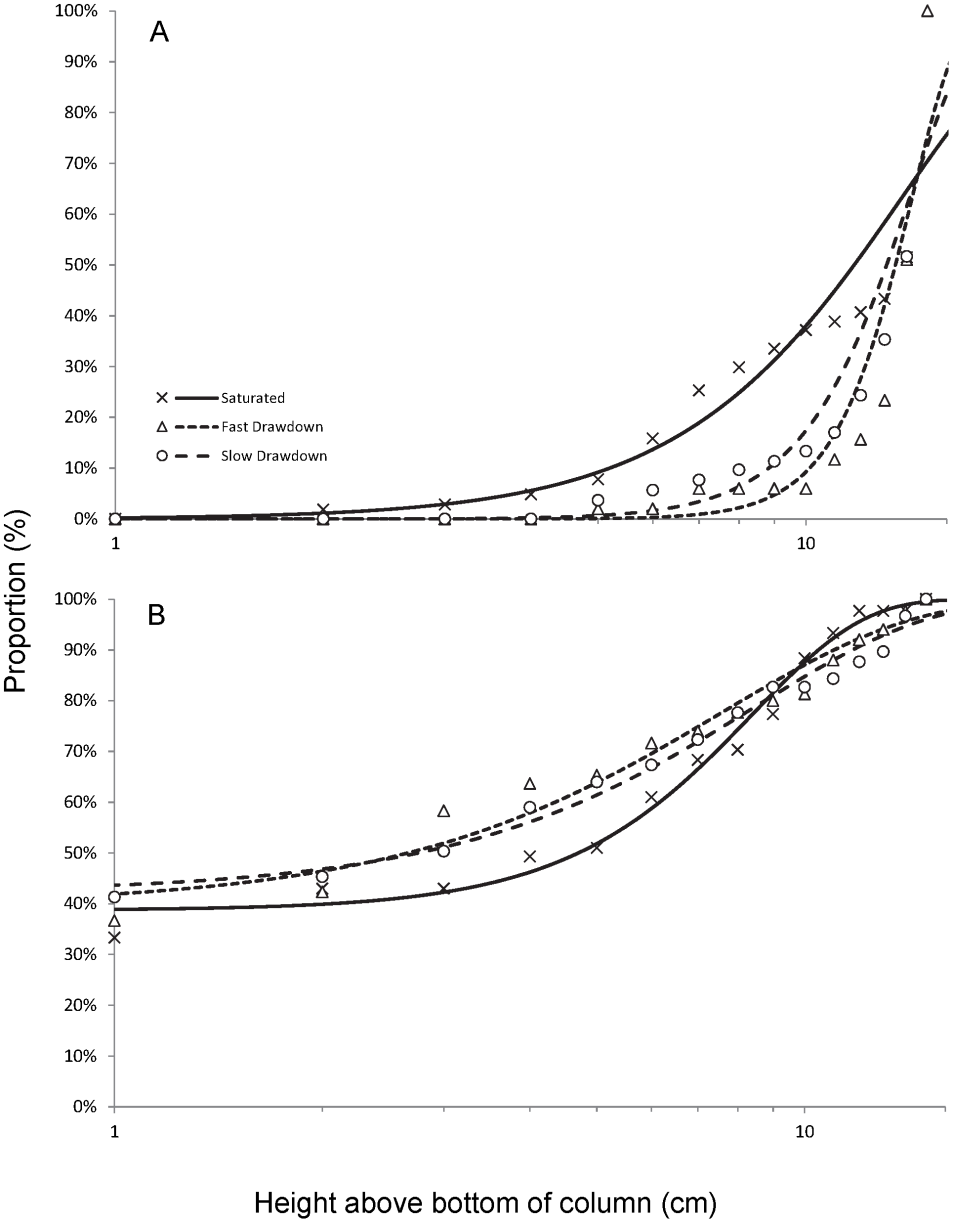
Cumulative depth distributions of copepods in saturated, fast and slow drawdown experiments. Comparison of depth distributions from A - the Wellington alluvium copepods and B - the Budderoo swamp copepods in sediment columns; zero depth refers to the base of the column.

In the saturated columns, the copepods were not equally distributed over depth but were most abundant in the top 4 cm from 12–16 cm (62±27%) and least abundant (3±4%) in the bottom (0–4 cm) of the column (Fig. 3A). After slow drawdown, most of the copepods were observed in the unsaturated zone (84±5%), some in the capillary fringe (9±3%) and even fewer (7±3%) below the water table (Fig. 3A). The distribution was similar following the fast drawdown. In the fast drawdown treatments, 88±6% of the Copepoda were observed in the unsaturated zone, with similar proportions in the capillary fringe (6±0%) and below the water table (6±6%).

**Figure 4:**
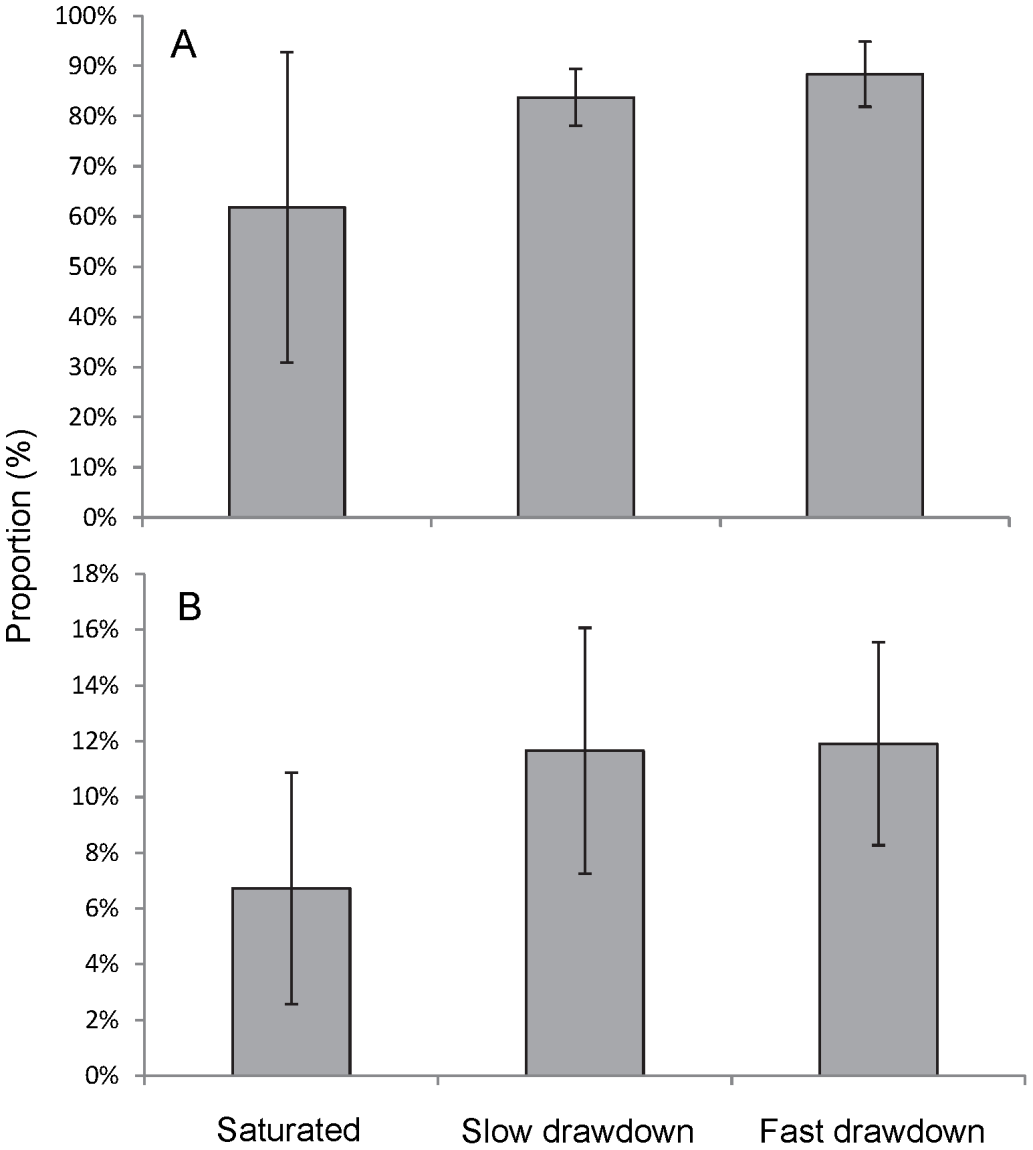
Mean (±95% CI) proportion of copepods in the top four centimetres of the sediment. Comparison of results on mean proportion of copepods from A - the Wellington alluvium and B - the Budderoo swamp occurring in the saturated and drawdown columns; in the latter the top four centimetres represents the unsaturated zone in the fast and slow drawdown columns and shows the proportion of animals stranded above the water table as the water level is lowered.

There was no significant difference in the overall distribution of copepods between the three treatments (p = 0.065, Fig. 3A). Similarly, there was no significant difference (p = 0.243) in the proportion of copepods in the upper most 4 cm of the columns (12–16 cm) following drawdown (Fig. 4A). Generally, the abundance of copepods decreased with depth below water table.

#### Budderoo Swamp Copepods

The relative abundance of copepods in the saturated columns increased with depth below the water table. We recorded 7±7%, 25±4%, and 68±36% of the organisms in 16-12 cm, 12-8 cm, and 8-0 cm, respectively (Fig. 3B). In the slow drawdown treatments, the relative abundances of copepods also increased with depth. In the unsaturated zone and the capillary fringe 16±5% and 12±8% of the organisms were found, respectively (Fig. 3B). On average, 72% of the organisms were present below the water table. For the fast drawdown, 12±6% of the copepods remained in the unsaturated zone, 14±6% in the capillary fringe, and 74% of the organisms stayed below the water table.

There was no significant difference in the distribution of copepods among the three treatments (p = 0.994, Fig. 3B). There was also no significant difference (p = 0.536, Fig. 4B) in the proportion of copepods in the upper 4 cm of the columns between the three treatments.

### Desiccation Experiments

#### Syncarids

**Figure 5:**
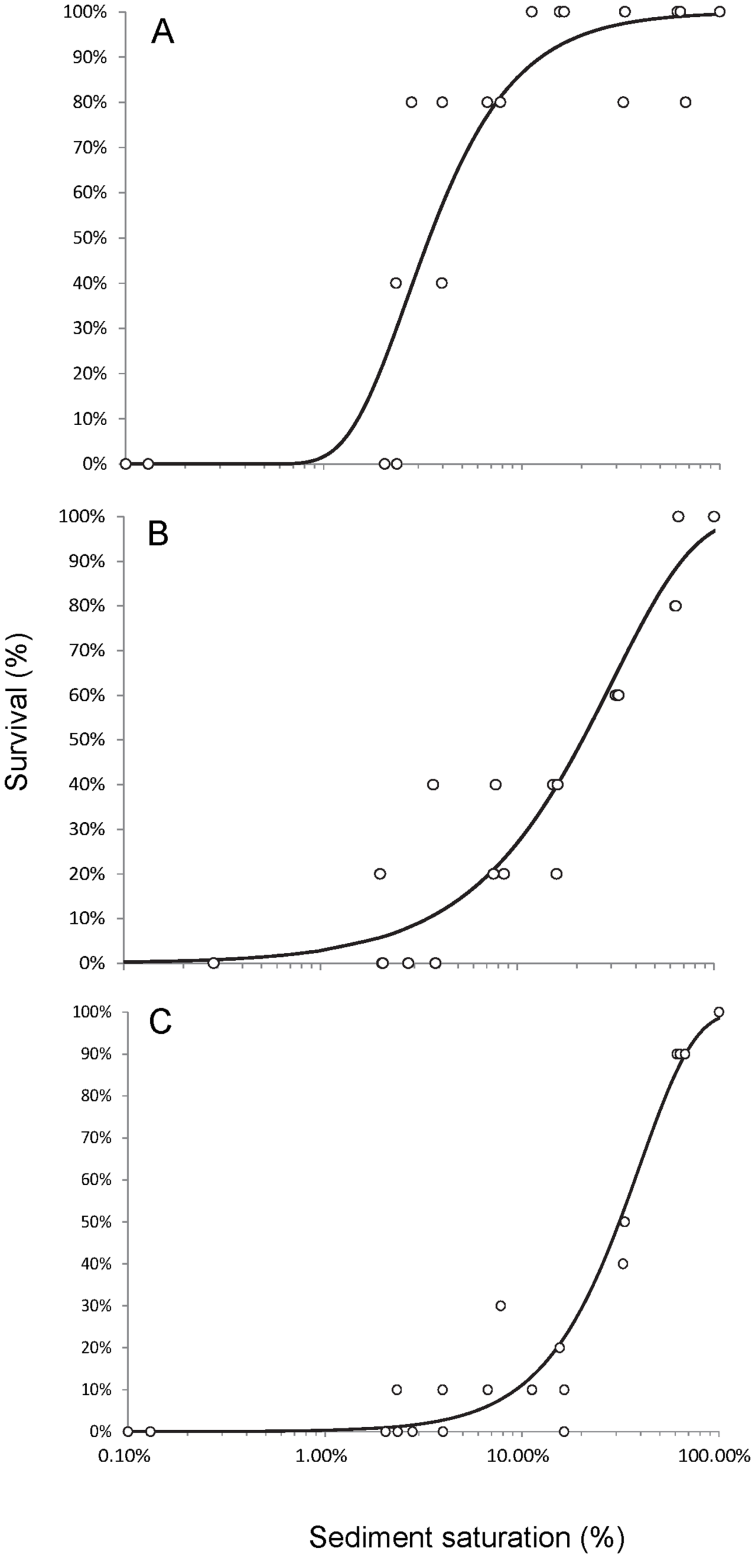
Survival rate of stygofauna depending on the sediment saturation level. Response of A - the Wellington syncarids, B - the Wellington copepods and C - the Budderoo swamp copepods to decreased sediment saturation for 48 h.

We found little effect of the water content changes on the survival of the syncarids down to saturation levels of 6% (Fig. 5A). Here, still 87% (±11%) were alive after 48 h exposure to these hydraulic conditions. The survival rate dropped steeply to 67% (±23%) and 13% (±23%) at 3% and 2% saturation level, respectively. At completely dry conditions all organisms were dead. For the syncarids, the 48 h LSL_50_ was 3.4% saturation, with 10% of the population affected at 12.5% (Table 1). There was 90% mortality in the population after 48 h at 1.5% saturation (Table 1).

**Table 1:** Lethal saturation level values affecting 90, 50 and 10% of the population.

	LSL_90_	LSL_50_	LSL_10_
Wellington Syncarida	0.015 (−0.002– 0.032)	0.034 (0.011–0.057)	0.125 (−0.040– 0.290)
Wellington Copepoda	0.093 (−0.017–0.205)	0.312 (0.130– 0.494)	0.676 (0.120– 1.15)
Budderoo Copepoda	0.035 (−0.024–0.094)	0.214 (0.054– 0.375)	0.682 (0.044–1.32)

Values in parentheses indicate 95% confidence intervals.

#### Wellington copepods

The Wellington copepods were more susceptible to drying than were the syncarids. Although 90±0% of the copepods were still alive under 63% of saturation, only 47±10% and 17±5% were alive at saturation levels of 33% and 6%, respectively (Fig. 5B). The 48 h LSL_50_ was 31.2%. Ten percent of the population were affected at 67.6% saturation and 90% mortality occurred at 9.3% (Table 1).

#### Budderoo Swamp copepods

The Copepoda from the Budderoo swamp responded to drying in a similar way as those from the Wellington alluvium. At 66% saturation, 87±12% of the organisms were still alive (Fig. 5C). Only one third of the Copepoda (33±12%) were alive after 48 h at saturation levels of 16%. The 48 h LSL_50_ was 21.4% (Table 1). Ten percent of animals were dead at saturation of 68% and 90% were dead at 3.5% (Table 1).

## Discussion

Our simulated lowering of water tables had significant impacts on groundwater fauna. Fauna became stranded as water tables declined. Our desiccation experiments show that the reduction in water content of the sediments associated with such water table lowering is likely to result in mortality of the stranded fauna.

In the fully saturated sediment columns, the distribution of animals differed markedly between taxa. For the syncarids and the swamp copepods, a large proportion of the animals were found towards the bottom of the column whereas for the Wellington copepods, the majority of animals were found close to the surface, with abundance decreasing with depth. Although the abundance of fauna generally decreases with depth below the water table [10], [32], Datry et al. [9] found different abundance-depth relationships in different taxa. In their study, the abundance of epigean taxa was higher near the water table compared to hypogean taxa which were generally found at greater depths. Interestingly, our results suggest the opposite. Among the copepods, the truly stygobitic taxon was most abundant at the surface and the stygophilic (potentially epigean) taxon most abundant at depth. Our centimetre-scale examination of laboratory columns is much finer than the metre-scale examination of *in situ* piezometers (up to 4 m below the water table) used by Datry et al. [9], and the differences observed may reflect the different scales examined, or indeed, different taxa could be adapted to depth-specific ecological niches, such as is known from marine systems [33]. However, the driving factors for stygofauna distribution are mainly related to nutrient availability and not just to pressure changes below the water table, which, over the small scale we have examined, is likely to be negligible.

In fine, centimetre-scale laboratory experiments [18], the distribution of copepods and amphipods followed that of the Wellington copepods, being higher in abundances closer to the water table. Similarly, the abundance of fauna, mainly Copepoda, Nematoda and Oligochaeta, in sand filters of drinking water production systems decreased with increasing filter depth down to 60 cm [34]. The occurrence of depth-related faunal distributions, even on the centimetre scale, emphasises the vulnerability of fauna in the vicinity to the water table to even small changes in water table depths.

The habitat from which the swamp copepods were collected is constantly saturated to the soil surface [35]. Despite small declines in the water table, the capillary forces of the highly organic surface layers ensure saturation to the surface (Hose & Stumpp unpub. data). Accordingly, the copepods from the swamp may not be accustomed to and therefore may be sensitive to fluctuations in the water table. This may explain why they were the taxon most susceptible to water level changes (i.e., with the greatest proportion of the population stranded) compared to the organisms from the Wellington site. The Wellington alluvial aquifer responds quickly to changes in water levels of the nearby river (up to ±9 m) and therefore, organisms could well be adapted to dynamic water level conditions.

The fate of groundwater fauna in response to water level changes may relate closely to their mode of locomotion too, i.e., if they are swimmers or crawlers. Crawlers can actively hold on to the sediment (like the syncarids) compared to swimmers (like the copepods) who prefer to be in open water spaces. We observed that the syncarids were fast crawlers and could move quickly through the pores of the sediment columns and in the petri dishes of the desiccation experiments. The change in the distribution of the syncarids before and after drawdown, in particular the greater proportion of syncarids found at the bottom of the fully saturated column, was not as we expected. We consider that this result is partly due to their mobility, which could enable them to move progressively downward ahead of the water column as we removed the sediment for analysis at the conclusion of the experiment. However, the fact that some syncarids were trapped in the unsaturated sediments suggests that their mobility in low saturation conditions may be limited, and that they were unable to detect the rapid decline in water levels (relative to the desiccation experiments) until such time as it was difficult to move or they were isolated. In the desiccation experiments, the rate of removal of water was much lower (0.03 m/d) than in the column experiments (2.6 m/d) and the syncarids were able to move to the bottom of the dish where water pooled as it dried. Consequently, the syncarids appear quite tolerant to drying, however, this may reflect more their ability to move to available water rather than a tolerance for dry conditions.

Despite the water table being lowered 8 cm and creating a 4-cm zone of unsaturated sediment, there was no significant difference in the distribution of copepods between treatments. This was the case for both copepod taxa. Copepods did not appear to move downwards with the receding water table and so were stranded in the unsaturated zone. In similar experiments in which water levels within 6–cm tall sediment tubes were lowered at a rate of 1.2 m/d, cyclopoid copepods moved downwards with the declining water table [19]. In contrast, amphipods did not move downwards and so had a similar vertical distribution to those in the saturated control columns [19]. It is unknown whether groundwater fauna can sense and adapt to a change in water flow such as is known from the hyporheic zone [18] or experiments simulating declining water levels in benthic surface water systems. For the latter it was shown in laboratory experiments that three amphipod species had mechanisms, such as horizontal migration in the water phase and vertical burying into the sediment, to survive drying surface water conditions [36].

The water saturation level in the columns after drawdown and survival of organisms in the desiccation experiments, suggest that severe impacts are likely for fauna stranded in the unsaturated zone. The saturation level in the unsaturated sediments (12–16 cm) was (on average) 32% which, based on the desiccation results, will cause around 50% mortality to animals. With over 80% of the copepods from the Wellington alluvium occurring in the unsaturated zone of the columns (12–16 cm), mortality to over 40% of the total population may be expected. The equivalent expected total mortalities for the Budderoo swamp copepods and the Wellington syncarids are smaller (around 5% and 1%, respectively). We found large differences in the LSL_50_ for syncarids and the copepods (Table 1). The LSL_50_ and LSL_10_ values for both copepod taxa were higher as previously reported, in which 50% of the copepods died at saturation levels of 10% and 10% died at saturation levels of about 55% [19]. Interestingly, Tomlinson [19] found no significant correlation between water content and survival, which varied between 40 and 100% in experiments with 20 to 100% saturation.

While we have considered here the impact of water level change *per se*, we have assumed that any fauna that can migrate with the water table will survive, but this may not always be the case. We assume that for field conditions changes in water depth may force fauna to move to sedimentary layers of different physical or chemical composition that may be suboptimal, creating a situation of habitat loss. This is indeed a topic for further examination. Further, here we have simulated an alluvial sediment, characterised by a large number of regular and large pore spaces. How changes in this system can be related to other aquifer types, such as karstic and fractured rock, needs to be tested. The different pore systems and hydraulic properties of these aquifer types make it difficult to translate the findings of our drawdown experiments, but where changes in water level lead to a reduction in saturation of the aquifer matrix, mortality of fauna, such as we have shown in the desiccation experiments, is likely, irrespective of aquifer type.

In this study we have shown using in-vitro experiments that changes to groundwater levels may pose a significant threat to the survival of groundwater biota, particularly in coarse sediments simulated here. It is likely that in finer sediments that hold water more readily, the impact to fauna may not be so great. However, with our drying period potentially at the lower end of those that might be expected under field conditions, significant risks to groundwater fauna remain.

The results of this work provide new understanding of the response of fauna to water table drawdown under controlled conditions. With immense and growing pressure on groundwater resources globally [11], such knowledge is timely and further research is required to transfer the knowledge from in-vitro experiments to field scales and study how water table changes might affect the survival of organisms and even biodiversity. Preserving the biodiversity of groundwater ecosystems is essential to maintain the ecosystem services these ecosystems provide. With improved understanding of how water level changes influence biota, targeted strategies to better manage groundwater abstraction and maintain groundwater biodiversity may be developed.

## Supporting Information

Appendix S1 **Mean proportion (± standard deviation) of organisms under different treatments across depth in the drawdown experiments.**
(XLS)

Appendix S2 **Mean proportion (± standard deviation) of organisms survived at different saturation levels in the desiccation experiments.**
(XLS)
